# Plasma Fibrinogen-Like 1 as a Potential Biomarker for Radiation-Induced Liver Injury

**DOI:** 10.3390/cells8091042

**Published:** 2019-09-06

**Authors:** Na-Kyung Han, Myung Gu Jung, Ye Ji Jeong, Yeonghoon Son, Su Chul Han, Seungwoo Park, Young-Bin Lim, Yoon-Jin Lee, Sung-Ho Kim, Su Cheol Park, Hae-June Lee

**Affiliations:** 1 Division of Radiation Biomedical Research, Korea Institute of Radiological & Medical Sciences, Seoul 01812, Korea (N.-K.H.) (M.G.J.) (Y.J.J.) (Y.S.) (Y.-B.L.) (Y.-J.L.); 2 Primate Resource Center, Korea Research Institute of Bioscience and Biotechnology (KRIBB), Jeonbuk 56216, Korea; 3 Division of Medical Radiation Equipment, Korea Institute of Radiological & Medical Sciences, Seoul 01812, Korea (S.C.H.) (S.P.); 4 College of Veterinary Medicine, Chonnam National University, Gwangju 61186, Korea; 5 Department of Internal Medicine, Korea Cancer Center Hospital, Korea Institute of Radiological and Medical Sciences, Seoul 01812, Korea

**Keywords:** FGL1, radiation toxicity, liver, plasma, biomarker

## Abstract

Liver damage upon exposure to ionizing radiation, whether accidental or because of therapy can contribute to liver dysfunction. Currently, radiation therapy is used for various cancers including hepatocellular carcinoma; however, the treatment dose is limited by poor liver tolerance to radiation. Furthermore, reliable biomarkers to predict liver damage and associated side-effects are unavailable. Here, we investigated fibrinogen-like 1 (FGL1)-expression in the liver and plasma after radiation exposure. We found that 30 Gy of liver irradiation (IR) induced cell death including apoptosis, necrosis, and autophagy, with fibrotic changes in the liver occurring during the acute and subacute phase in mice. Moreover, FGL1 expression pattern in the liver following IR was associated with liver damage represented by injury-related proteins and oxidative stress markers. We confirmed the association between FGL1 expression and hepatocellular injury by exposing human hepatocytes to radiation. To determine its suitability, as a potential biomarker for radiation-induced liver injury, we measured FGL1 in the liver tissue and the plasma of mice following total body irradiation (TBI) or liver IR. In TBI, FGL1 showed the highest elevation in the liver compared to other major internal organs including the heart, lung, kidney, and intestine. Notably, plasma FGL1 showed good correlation with radiation dose by liver IR. Our data revealed that FGL1 upregulation indicates hepatocellular injury in response to IR. These results suggest that plasma FGL1 may represent a potential biomarker for acute and subacute radiation exposure to the liver.

## 1. Introduction

The tolerance of healthy tissue to radiation therapy (RT) limits the dose of radiation that can be delivered during the treatment of malignancies [1]. Despite continuing technical developments in RT, both the acute and delayed side effects of radiation in healthy tissue remain significant obstacles to cancer treatment. Thus, there is an increasing need to identify typical tissue markers able to predict an unusual risk of complications during the therapeutic phase, diagnose radiation-related toxicity, and be rapidly applied in the case of bioterror for tissue-specific biodosimetry [2,3]. However, few clinically useful radiation-related biomarkers have been identified. 

The liver is one of the organs critically exposed to the risks associated with RT for many abdominal and lower thoracic tumors such as the gastric, distal esophagus, lower lung and breast, bile duct, pancreas, and whole abdomen [4,5]. Indeed, targeted liver irradiation also causes cell damage and liver dysfunction [5]. In patients, the resulting liver injury, termed radiation-induced liver disease (RILD), occurs from two weeks to six months after RT [6]. Liver radiation injury is histologically characterized by a loss of parenchymal hepatocytes, distortion of the lobular architecture, venous obstruction, and sinusoidal congestions accompanied by clinical symptoms such as fatigue, ascites, and elevated liver enzymes [7]. Hepatocytes are the principal parenchymal cells carrying out most of the metabolic functions of the organ and constitute about 80% of the liver population. It is well known that hepatocellular death is the ultimate driver of the progression of other liver diseases and the development of liver fibrosis, cirrhosis, and hepatocellular carcinoma (HCC) [8]. Hence, it is necessary to investigate hepatocellular responses to radiation and identify reliable biomarkers to detect hepatic injury. 

Fibrinogen-like 1 (FGL1), also termed hepatocyte-derived fibrinogen-related protein 1 (HFREP1) or hepassocin, constitutes a hepatocyte-secreted protein that was initially cloned from and found to be over-expressed in human HCC [9,10]. FGL1 contains a fibrinogen-related domain in its C-terminal portion but lacks three functional domains of platelet binding site, crosslinking region, and thrombin-sensitive site [9]. The exact role of FGL1 in the liver is controversial. It has been reported that exogenous FGL1 promotes the proliferation of normal hepatocytes, stimulates hepatocyte proliferation in vivo, and prevents the rat liver injury induced by d-galactosamine and CCl_4_ [11,12]. Paradoxically, FGL1 has also shown a suppressive effect on the growth of hepatocellular carcinoma cells [13,14]. A recent study also demonstrated that FGL1 could regulate lipid metabolism and energy utilization owing to its structural similarity with angiopoietin-like factors [15]. Additionally, it has been reported that FGL1 has a critical role in the development of non-alcoholic liver disease [16]. However, the impact of FGL1 on radiation-induced liver injury remains obscure.

This study investigated the potential for plasma FGL1 levels to serve as a predictive marker for radiation-induced hepatic injury. We found that FGL1 was upregulated in the liver and plasma during irradiation-induced hepatocellular damage. Plasma FGL1 levels appear to have potential as a biomarker representing radiation response in a dose-dependent manner. 

## 2. Experimental Section

### 2.1. Animal Experiments

Male BALB/c mice were obtained from Orient Inc. (Seoul, South Korea) at 7 weeks of age (average body weight, 18.2 ± 2.1 g) and acclimated for 1 week prior to each experiment. Animals were kept under normal conditions of free access to water and food. Total 154 mice were randomly assigned to each experiment: (1) the study to establish the radiation liver injury mouse model; 5 mice for sham- or irradiated group, (2) FGL1 measurement by time course; each 5 mice for liver IR and total body IR at 0, 0.5, 1, 2, 3, 5 and 7 days after irradiation, (3) radiation dose-dependency test; 5 mice for 0, 5, 10, 20 and 30 Gy, (4) comparison test of whole liver IR (15 and 30 Gy) and focal liver IR (45 Gy); 10 mice for control and each 13 mice for irradiated group. The studies were carried out under the guidelines for the use and care of laboratory animals and were approved by the Institutional Animal Care and Use Committee of the Korea Institute Radiological and Medical Science (#KIRAMS 2014-0045 and #KIRAMS 2017-0007).

### 2.2. Liver Imaging

For focused radiation beam targeting of the mouse liver, computed tomography (CT) images of anesthetized mice were acquired using Micro-CT imaging, which was performed with a small animal micro-ionizing radiation (IR) instrument (X-RAD 225Cx, Precision X-ray Inc., North Branford, CT, USA). Scan range of the liver was defined via the software (PiotXRAD, ver. 1.14.5, North Branford, CT, USA). Acquired images from Micro-CT were delivered to the small animal Smart-Plan radiotherapy planning system (version 1.5.0, Precision X-ray Inc., North Branford, CT, USA) to create, evaluate, and deliver irradiation. 

### 2.3. Irradiation

Each mouse was anesthetized with tiletamine/zolazepam (Zoletil 50^®^, Virbac Korea, Seoul, Korea) for irradiation. Using the guidance software utility of the XRAD-SmART, cone-beam CT images were co-registered by manual fusion; the isocenter of the liver was identified and aligned with the central axis of the beam. Mice were irradiated using the XRAD-SmART system at 225 kV (peak) with 13 mA X-ray beams at a dose rate of 3.53 Gy/min using additional filtering of 0.3 mmCu, with irradiation at 305.3 mm source-to-isocenter distance. 15 or 30 Gy of whole liver IR was applied to the lateral abdominal region of mice bidirectionally (each 90, 270 degrees with 7.5 Gy and 15 Gy, respectively) using a 10 × 10 mm^2^ square collimators. Focal hepatic IR was administered into the right abdominal region of mice in 4 directions with 11.25 Gy (each 11.25 Gy, 0, 60, 240, and 270 degrees) using a 3 × 3 mm^2^ square collimator.

For total body irradiation, mice exposed to 10 Gy of IR using an X-RAD 320 system (Precision X-ray, Inc., North Branford, CT, USA) at 250 kV and 10 mA with 420 mm of aluminum shielding, resulting in a dose rate of 2 Gy/min. The mice were placed in an irradiation field with a 20 cm × 20 cm size that included entire body. Control mice were treated in the same manner but were not exposed to the X-ray beams (sham-irradiated).

### 2.4. Sample Preparation

After animals were sacrificed, plasma was prepared from blood, and organ tissues were immediately fixed in 10% neutralized formalin or frozen in liquid nitrogen and stored at −80 °C for Western blotting, quantitative real-time PCR, or enzyme-linked immunosorbent assay (ELISA).

### 2.5. Histopathological Analysis

The fixed liver tissue samples were processed and paraffin embedded. Three µm thick sections were obtained, dewaxed, and then rehydrated. Hematoxylin and eosin staining was conducted using a standard protocol. For immunohistochemistry, endogenous peroxidase was blocked with 0.3% H_2_O_2_ in absolute methanol for 15 min at room temperature. For antigen retrieval, sections were placed in a citric buffer (pH 6.0) that was heated in an autoclave for 20 min. Non-specific immunoglobulin binding was prevented by incubating the section in blocking solution for 30 min. The sections were incubated at room temperature with diluted FGL1 antibody (1: 100, Cat# 16000-1-AP, Proteintech, Rosemont, IL, USA) and 8-hydroxy-2′-deoxyguanosine (8-OHdG) antibody (1:2,500, Cat#ab62623, Abcam, Cambridge, CA, UK) and washed with phosphate buffered saline containing 0.1% Triton X-100 (2 × 4 min). Incubation with the corresponding secondary antibody and the peroxidase antiperoxidase complex was performed for 30 min at room temperature. The immunoreactive sites were visualized using 3,3′-diaminobenzidine (0.1%) and hydrogen peroxide solution (0.03%). Terminal deoxynucleotidyl transferase dUTP nick-end labeling (TUNEL) staining was conducted using the ApopTag® Peroxidase In Situ Apoptosis Detection Kit (Millipore, Billerica, MA, USA) according to the manufacturer’s instructions. For Sirius red staining, sections were incubated with 0.1% Sirius red (Sigma-Aldrich, St. Louis, MO, USA) solution dissolved in aqueous saturated picric acid for 1 h, washed in acidified water (0.5% hydrogen chloride), dehydrated, and mounted. Hematoxylin was used as the counterstain. Images were obtained using a BX-53 microscope equipped with a CCD DP73 digital camera (Olympus, Tokyo, Japan).

### 2.6. Western Blotting

Cells and liver tissue were collected and lysed with RIPA buffer (Intron Biotechnology, Seoul, Korea) containing complete protease inhibitor cocktail (Roche Diagnostics, Indianapolis, IN, USA), and protein concentration was determined by a protein assay (Bio-Rad, Hercules, CA, USA). A 30 μg aliquot of protein sample was size-fractionated by electrophoresis and then transferred to a nitrocellulose membrane. The membranes were immunoblotted with primary antibodies and horseradish peroxidase-conjugated anti-mouse IgG. The immunoblotted proteins were visualized using an enhanced chemiluminescent system (Amersham Biosciences, Arlington Heights, IL, USA). The primary antibodies were purchased from the following sources: anti-FGL1, anti-proliferating cell nuclear antigen (PCNA) (1:1000, SC-56; Santa Cruz Biotechnology, Dallas, TX, USA), anti-alpha smooth muscle actin (α-SMA) (1:1000, Cat# ab5694, Abcam, Cambridge, CA, UK), anti-fibroblast-specific protein-1 (FSP-1) (1:1000, Cat# ab27957, Abcam), anti-cleaved caspase-3 (1:500, Cat# 9661, Cell Signaling Technology, Boston, MA, USA), anti-LC3 (1:5000, Cat# NB100-2220, Novus Biologicals, Littleton, CO, USA), and anti-β-actin (1: 3000, Cat# A2228, Sigma-Aldrich). 

### 2.7. Cell Culture and Reagents

Human hepatocyte (HH) cells were obtained from Sciencell Research Laboratories (Carlsbad, CA, USA). They were cultured in hepatocyte medium supplemented with 5% fetal bovine serum (Sciencell), 1% penicillin and streptomycin (Sciencell), and 1% hepatocyte growth supplement (Sciencell). The cells were grown at 37 °C in 5% CO_2_. One day before irradiation with a dose of 0–15 Gy using by ^137^Cs as a radiation source (GC3000, Atomic Energy of Canada, Mississauga, Canada), HHs were seeded. The cells were harvested for Western blotting or quantitative real-time reverse transcription-PCR at the indicated time point.

### 2.8. Quantitative Real-Time Reverse Transcription-PCR (RT-qPCR)

For analysis of mRNA expression, RNA was isolated from cell lines using TRIzol (Qiagen, Valencia, CA, USA) following the manufacturer’s instructions. RNA concentration was determined using an Ultrospec 2000 (GE Healthcare Life Sciences, Buckinghamshire, UK). cDNA was synthesized using the cDNA Synthesis Master Mix (GenDEPOT, Barker, TX, USA) with random hexamer and OligodT. RT-qPCR analysis was performed using the CFX96TM Real-Time PCR System (Bio-Rad) using 100 ng of cDNA product and Sybr Green PCR Master mix. The relative expressions of genes were normalized to those of the housekeeping gene encoding glyceraldehyde 3-phosphate dehydrogenase (GAPDH). For human FGL1 gene assessment, the primers were as follows: FGL1 forward: 5′-CTGGAGATTCCCTTGCGG-3′, FGL1 reverse: 5′-GTTTTAGCCGTGTAGGGG-3′, GAPDH forward: 5′-CGAGATCCCTCCAAAATCAA-3′, and GAPDH reverse: 5′-CCTTCTCCATGGTGGTGAA-3′.

### 2.9. ELISA

For the analysis of secreted FGL1 protein, secreted mouse, and human FGL1 levels were measured using the commercially available mouse and human fibrinogen-like protein 1 ELISA kit (Cusabio Biotech, Wuhan, China). Manufacturer protocols were followed, and samples were measured in duplicate.

### 2.10. Statistical Analysis

All values are presented as the means ± standard deviation (SD). Data were analyzed using analysis of variance (ANOVA) with a Dunnett’s post hoc test for multiple comparisons or student *t*-test for two groups. *p*-values < 0.05 were considered to indicate statistical significance.

## 3. Results

### 3.1. Up-Regulation of FGL1 in Mouse Liver by Irradiation

#### 3.1.1. Association between FGL1 and Radiation-Induced Liver Injury 

**Figure 1:**
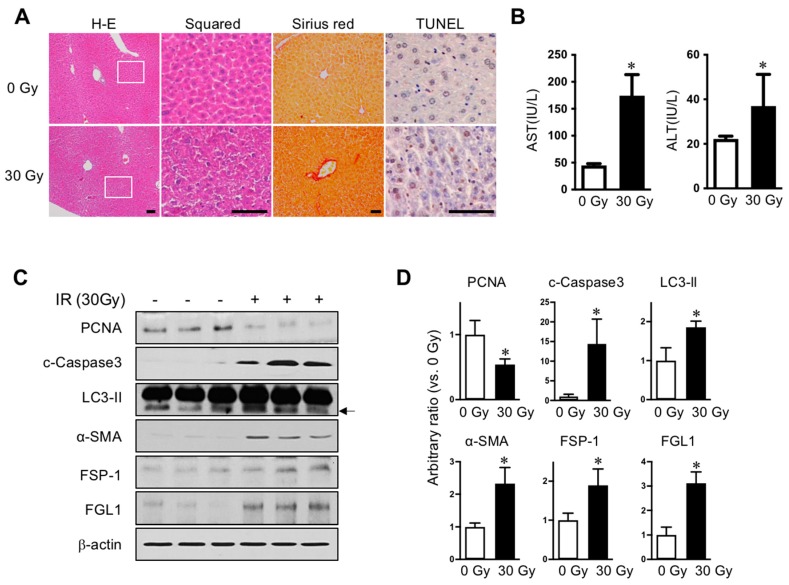
Radiation-induced liver injury and FGL1 expression. (**A**) Representative liver histology showing hematoxylin and eosin (H–E), Sirius red, and TUNEL assay. Inserts show higher magnification. Scale bar: 100 μm. (**B**) Serum alanine transaminase (ALT)/aspartate transaminase (AST) level (n = 5 mice/group). Mean ± SD results are graphed (unpaired two-sample Student’s t-test, * *p* < 0.05 compared to 0 Gy control). (**C**) Representative images and (**D**) quantitative data show alterations of PCNA, cleaved caspase-3, LC3-II, α-SMA, FSP-1 and FGL1. β-actin was used as the loading control. Western blotting was performed using liver tissues harvested at 7 days after IR. Data represented Mean ± SD (n = 3, * *p* < 0.05 vs. non-irradiated control).

We established radiation-induced liver injury model to compare the liver subjected to radiation injury with normal liver. To avoid exposure to other internal organs adjacent to the liver, especially the intestine, we applied 30 Gy of liver IR encompassing over 80% of the liver volume, but rarely including the intestine, stomach, or heart by Micro-CT scanning prior to the irradiation using image-guided small animal radiotherapy (XRAD-SmART). Radiation is known to cause liver injury characterized by cell death and fibrotic changes in rodents [7]. In histopathological results, 30 Gy radiation led to destroyed parenchymal structures, fibrotic changes, and apoptotic death in the liver at 7 days following liver irradiation, as shown by hematoxylin and eosin and Sirius red staining, and TUNEL assays, respectively (Figure 1A). In the liver-injured mice, aspartate transaminase and alanine transaminase levels in plasma were significantly increased (Figure 1B). Consistent with this, FSP-1, cleaved caspase-3, and LC3-II were increased and PCNA was decreased in irradiated liver compared to that in un-exposed control liver, assessed using Western blot analysis (Figure 1C,D). Moreover, expression of FGL1 protein significantly increased with similar pattern to other injury signals, which suggested a potential role for FGL1 in indicating liver injury induced by IR. 

#### 3.1.2. Up-Regulation of FGL1 and 8-OHdG in the Liver at the Subacute Phase after IR

**Figure 2:**
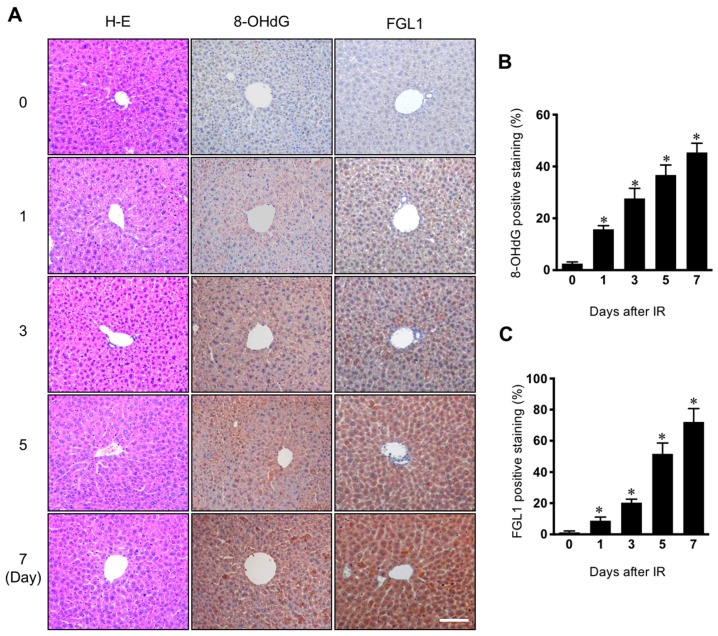
Expression of 8-OHdG and FGL1 in the liver following 30 Gy of whole liver irradiation. (**A**) Liver section stained at various days after IR. Hematoxylin-eosin (H–E), FGL1, and 8-hydroxy-2′-deoxyguanosine (8-OHdG) expression in the liver was determined by immunohistochemistry. Scale bar: 100 μm. (**B**,**C**) Quantification of the positive signal of FGL1 and 8-OHdG in a unit area at the indicated. Data represented Mean ± SD (n = 5, * *p* < 0.05 vs. the day 0 control).

Since it is well known that reactive oxygen species are a major cause of pathogenesis of normal tissue injury by irradiation [17], we investigated the association between FGL1 expression and oxidative stress in the radiation-induced liver injury model. In immunohistochemistry for FGL1 in liver tissue, FGL1 was rarely expressed in the cytoplasm of hepatocytes in non-irradiated control mice, whereas following 30 Gy of liver IR, FGL1 dramatically increased in the liver over time. We also conducted immunohistochemistry for 8-OHdG, a marker of oxidative damage, and observed increases in 8-OHdG expression in the liver (Figure 2A). Quantification of FGL1 positive staining and 8-OHdG positive regions showed a similar signature, increasing over time during the 7 days after IR (Figure 2B,C).

### 3.2. Up-Regulation of FGL1 in Human Hepatocytes Responding to Radiation 

#### 3.2.1. FGL1 Expression in HHs Following IR

**Figure 3:**
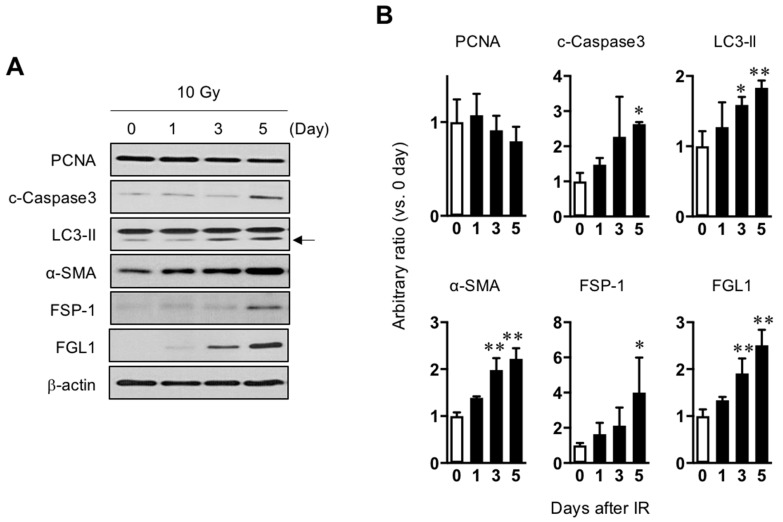
Association between radiation-induced hepatocyte damage and FGL1 expression. (**A**) Western blots analysis of lysates from hepatocytes treated with 10 Gy of radiation for PCNA, cleaved caspase-3, LC3-II, α-SMA, FSP-1, and FGL1 at the indicated days. (**B**) The indicated protein expression is represented as relative values compared with the non-irradiated control in the graph. Data represented Mean ± SD (n = 3, * *p* < 0.05 or ** *p* < 0.01 compared to the day 0 control).

As FGL1 is known to be derived from human hepatocytes (HHs), HHs were exposed to 10 Gy of radiation and then observed radiation injury signals for 5 days after IR. Consistent with the results of mouse liver (Figure 1C,D). PCNA, a proliferation marker, showed a gradual decrease until 5 days whereas α-SMA and FSP-1 which are fibrotic signal markers, gradually increased until 5 days. In addition, apoptotic death-related cleaved caspase-3 and LC3-isoform II, a component of autophagosomes, was upregulated after IR. FGL1 protein was slightly increased after IR and significantly increased from 3 days post-IR (Figure 3 and Supplementary Figure S1). 

#### 3.2.2. FGL1 Upregulation by Radiation or Oxidative Stress from Hydrogen Peroxidase in HHs

**Figure 4:**
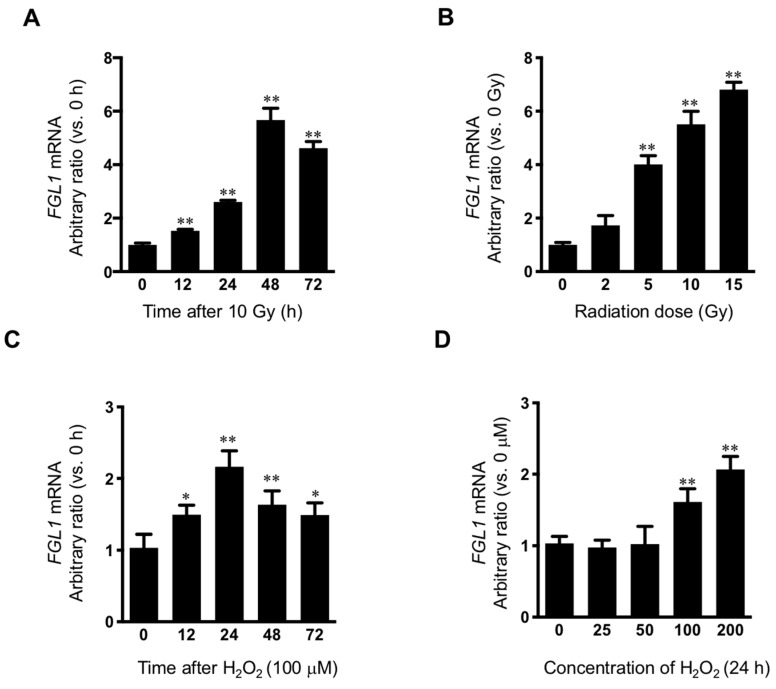
Induction of FGL1 mRNA in hepatocytes following irradiation and hydrogen peroxidase. (**A**) Human hepatocyte (HH) exposed to 10 Gy of IR and FGL1 mRNA expression was determined by RT-qPCR at the indicated time. (**B**) Radiation dose dependency of FGL1 mRNA expression were evaluated. (**C**) mRNA expression of FGL1 was measured in hepatocytes treated with 100 μM of H_2_O_2_ at different time points. (**D**) mRNA expression of FGL1 was measured in hepatocytes treated with different concentrations of H_2_O_2_ for 24 h. FGL1 mRNA expression were normalized to GAPDH and represented arbitrary ratio compared to non-treated control. Data represented Mean ± SD (* *p* < 0.05 and ** *p* < 0.01, n = 3).

Because marked FGL1 protein increase was observed at 3 days after 10 Gy of IR in HHs, *FGL1* mRNA was examined at 0, 12, 24, 48, and 72 h after 10 Gy of IR in HHs. *FGL1* mRNA was upregulated at 12 h and showed peak induction at 48 h after IR (Figure 4A). To confirm the radiation dose-dependence of *FGL1*, HHs were exposed to 0–15 Gy of radiation and mRNA was detected at 48 h after IR by RT-qPCR. *FGL1* mRNA was upregulated in a dose-dependent manner up to 15 Gy (Figure 4B). As FGL1 constitutes a secretory protein, we determined whether HHs secreted FGL1 into the culture medium. Secreted FGL1 was detected at 12 h and increased until 72 h after IR in the culture medium, as determined by ELISA (Supplementary Figure S2A). Furthermore, FGL1 in culture medium following different doses of radiation increased in an IR dose-dependent manner at 72 h after IR according to the ELISA assay (Supplementary Figure S2B). To determine the potential of FGL1 induction by reactive oxygen species in HHs, which are known to comprise a critical cause of radiation cell injury, hepatocytes were treated with hydrogen peroxidase (H_2_O_2_), and *FGL1* mRNA expression was analyzed. *FGL1* mRNA showed peak upregulation (>2-fold compared with the control) at 24 h with 100 μM of H_2_O_2_ (Figure 4C). Moreover, *FGL1* mRNA was upregulated at the concentration of over 100 μM of H_2_O_2_ (Figure 4D). These results are consistent with the induction of FGL1 and 8-OHdG by radiation in the liver tissue, as represented by the results of immunohistochemistry in Figure 2 and indicated that FGL1 upregulation may be mediated by oxidative stress from radiation and other stimuli. 

### 3.3. Plasma FGL1 as a Biomarker for Radiation Liver Injury

#### 3.3.1. FGL1 Expression in the Liver and Plasma Following Total Body Exposure

**Figure 5:**
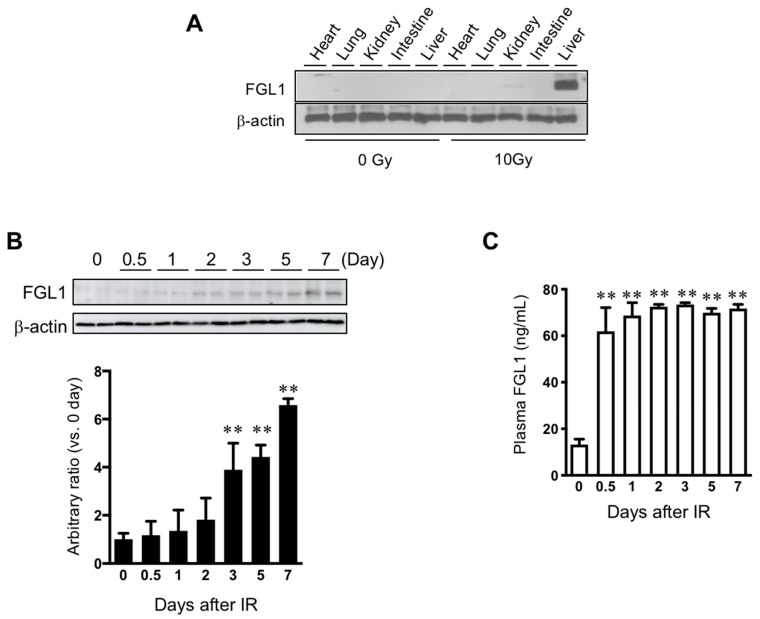
Expression of FGL1 in liver tissue and plasma following total body irradiation. (**A**) FGL1 expression of five internal major organs at 3 days after 10 Gy of total body irradiation. (**B**) Detection of FGL1 expression in total liver extracts by Western blotting and (**C**) in plasma by ELISA assay at days 0–7 following 10 Gy of total body irradiation. Data represent the means ± SD (** *p* < 0.01 compared to non-IR control, n = 4).

To determine the sensitivity of FGL1 to liver injury, 10 Gy of total body irradiation was performed. Measurement of FGL1 expression in major organs (heart, lung, kidney, intestine, and liver) showed the highest elevation of FGL1 levels in the liver at 3 days after TBI (Figure 5A). Also, we found no changes of expression of FGL1 in the pancreas by irradiation (Supplementary Figure S3). This indicated that the liver could contribute to an increase in the FGL1 levels in the plasma after TBI. In the liver tissue, FGL1 protein expression was significantly upregulated from 3 days after IR and remained detectable high levels until 7 days after IR (Figure 5B). FGL1 was detectable in plasma and sensitively responded to irradiation (Figure 5C). These results demonstrated that the possibility of FGL1 in the plasma as a novel biomarker at the acute and subacute phase of radiation liver injury when mice were exposed to total body irradiation. 

#### 3.3.2. FGL1 Expression in the Liver and Plasma Following Liver IR

**Figure 6:**
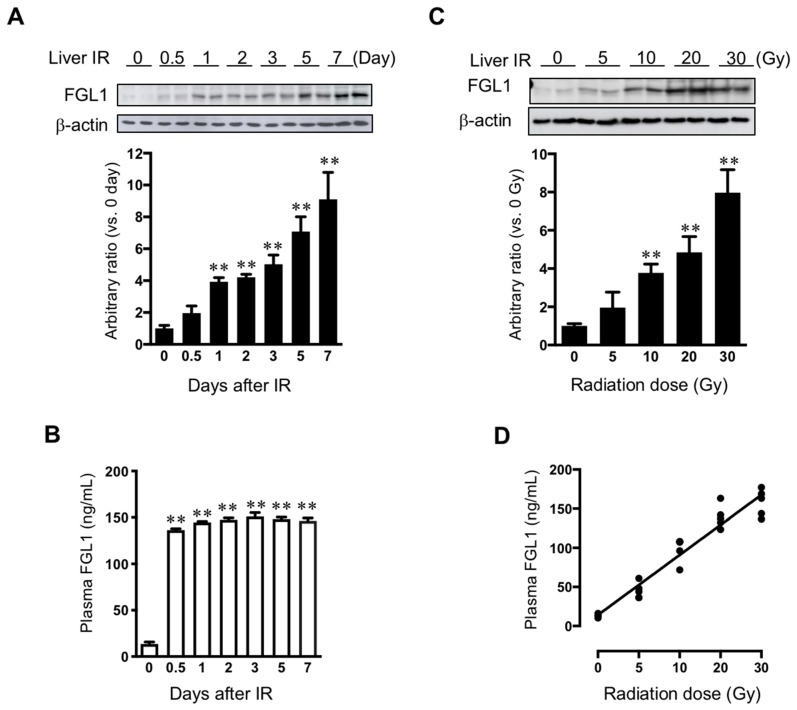
Expression of FGL1 in liver tissue and plasma following liver IR. (**A**) FGL1 expression in total protein extract from the liver and (**B**) mouse plasma at days 0–7 following 30 Gy of liver IR. (**C**) FGL1 induction in the liver and of mice following indicated radiation dose of liver IR at 3 days post-IR. Data represent the means ± SD (** *p* < 0.01, n = 5). (**D**) The relationship between in the plasma FGL1 concentration and radiation dose (*R^2^* = 0.93, *p* < 0.0001).

We performed Western blot assay for FGL1 in liver tissue harvested from 0 to 7 days after 30 Gy of liver IR. FGL1 protein in the liver tissue showed a slight increase at 0.5 day after IR. FGL1 in the liver tissue significantly increased from 1 day and then gradually increased over time with statistical significance (Figure 6A). Otherwise, plasma FGL1 started to increase after 12 h and increased until 2 days post-IR, then stayed high levels until 7 days after IR (Figure 6B). To further investigate the potential of FGL1 as a biomarker for liver injury, we examined FGL1 expression in the liver and plasma whether FGL1 has dose-dependent responses to radiation or not. We performed liver IR to mice with 0, 5, 10, 20, and 30 Gy and FGL1 protein in the liver tissue at 3 days following liver IR. FGL1 was detected following as low as 10 Gy in the liver tissue (Figure 6C). Interestingly, we can detect plasma FGL1 at 5 Gy by ELISA and plasma FGL1 increased in a dose-dependent manner. We found that radiation dose and plasma FGL1 showed a positive correlation (*R^2^* = 0.93, *p* < 0.0001) (Figure 6D). These data suggested that plasma FGL1 may represent a potential biomarker to reflect radiation dose-dependency to liver.

#### 3.3.3. Association between Plasma FGL1 and the Radiation Dose or Irradiation Field Size Relative to the Liver

**Figure 7:**
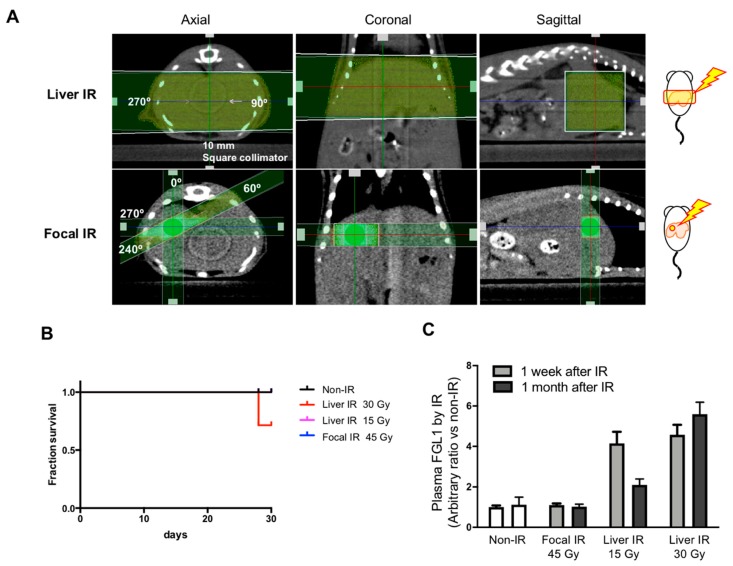
Measurement of FGL1 in mouse plasma following Liver IR or focal IR. (**A**) Schematics and representative photographs of mice exposed to whole liver IR and focal IR to the liver. Liver IR of 15 and 30 Gy was performed to the lateral abdominal region of mice bidirectionally (each 90, 270 degrees with 7.5 Gy and 15 Gy, respectively) using a 10 × 10 mm^2^ square collimator. Focal hepatic IR was administered to the right abdominal region of mice in 4 directions with 11.25 Gy (each 11.25 Gy, 0, 60, 240, and 270 degrees) using a 3 × 3 mm^2^ square collimator. (**B**) Detection of plasma FGL1 in mice at 7 and 30 days after IR in each experimental group was performed by ELISA (n = 4–7). Data represent the means ± SD. (**C**) 30 day-survival curves of the each group (n = 6–7).

Since hepatic injury is one of important toxicities when the whole liver is exposed to radiation up to 30-35 Gy [18,19], but is rare following whole liver exposure at a dose less than 15 Gy [20,21], we compared FGL1 levels in plasma according to irradiation technique. We applied 15 and 30 Gy of whole liver IR encompassing over 80% of the liver volume to the lateral abdominal region of mice using a 10 × 10 mm^2^ square collimator. In focal liver IR encompassing less 10% of the liver volume, 45 Gy of X-ray was applied to the liver using a 3 × 3 mm^2^ square collimator (Figure 7A). There were 2 mice out of 7 deaths in the group receiving 30 Gy of whole liver IR at 28th day (Figure 7B). 30 Gy of whole liver IR resulted in elevated post plasma FGL1 at 7 days and further increases at 30 days after IR whereas those of 15 Gy of whole liver IR was elevated at 7 days and then decreased at post 30 days. Plasma FGL1 in focal liver IR-treated mice was not different in age-matched non-irradiated control (Figure 7C). These results indicated that detection of FGL1 in plasma could depend on irradiated volume and dose and persisting of elevated plasma FGL1 during subacute phase could represent severe liver injury. 

## 4. Discussion

Radiation toxicity remains a challenging obstacle to therapeutic efficiency, including radiotherapy of the liver. Although many trials have aimed at finding a reliable biomarker to detect liver injury during RT, few molecular candidates have been reported with these having many limitations in application. For example, cleaved cytokeratin-18 represents a candidate marker for liver injury [22,23]. However, it has also been found in the blood of patients with sepsis [24] or rectal carcinoma [25]. Plasma LCN2 is currently reported to be an acute reactant that has radiation-specific expression [26]. However, LCN2 expression was found to be transitory (with a peak at 48–72 h followed by a sudden decrease to the basal level. Furthermore, LCN2 is well known as a biomarker for kidney injury [27]. Therefore, more sensitive biomarkers are needed to better predict radiation-liver toxicity.

In this study, the expression FGL1 in liver tissue or human hepatocytes following irradiation compared with those of normal individuals in order to identify a novel biomarker for radiation-induced liver injury. We demonstrated that radiation activated various signals including fibrosis (α-SMA, FSP-1), apoptosis (cleaved caspase-3) and autophagy (LC3-II) and these damage signals were well correlated with FGL1 expression in both in vivo and in vitro (Figure 3 and Figure 4). As FGL1 is a hepatocyte secreted protein, we examined plasma FGL1 expression response to radiation in order to investigate the potential of FGL1 as a biomarker for liver injury. We confirmed FGL1 increased in the liver tissue responded to in vivo liver injury coincident with in vitro results and could detect in the mouse plasma with radiation dose-dependency (Figure 1 and Figure 6). These results demonstrated that FGL1 is likely to constitute a stable and reliable biomarker reflecting liver injury via hepatocellular response to radiation. 

A recent study reported that FGL1 as a molecular candidate for radiation-induced lung fibrosis suggesting the involvement of radiation epithelial to mesenchymal transition using L132 human pulmonary epithelial cells [28]. Consistent with this previous report, irradiated HH showed upregulation of fibrotic/mesenchymal markers such as α-SMA and FSP-1 when FGL1 is elevated in Figure 3. However, we did not observe any alteration of FGL1 in the lung following 10 Gy of TBI, and it is different from result of Jin et al. that observed FGL1 up-regulation in fibrotic region of lung following 20 Gy of IR at 12 months after IR. Therefore, further studies will be needed to investigate the role of FGL1 based on molecular mechanism. Also, further biomedical studies including comparison FGL1 with other acute reactants for radiation liver injury such as plasma LCN2 will be useful to validate the potential as a liver biomarker.

In this study, we investigated the relationship between plasma FGL1 and dose-volume tolerance for radiation liver toxicity using whole liver IR- and focal liver IR-models. Historically, hepatic toxicity is one of the important toxicities when the whole liver is exposed to radiation of up to 30–35 Gy [18,19], although the radiation dose necessary to control most solid malignancies is approximately 50–70 Gy [20]. Thus, conventional RT targeting large volumes of the liver for the management of upper gastrointestinal and hepatobiliary malignancies may result in a high probability of radiation liver toxicity, known as radiation-induced liver disease (RILD). Stereotactic ablative radiotherapy (SABR) is a newly emerging treatment method to deliver a high dose to the target, utilizing either a single dose or a small number of fractions with a high degree of precision within the body [29]. Among the patients receiving conventional RT of 30–35 Gy, ~6–66% of patients present significant RILD [30,31,32]. This broad range of RILD incidence rate is related to the volume of irradiated livers, individual hepatic functional reserve and medical history [7]. However, it is reported that RILD after SABR occurs in fewer than 5% of cases [31]. One recent study reported SABR using 30–60 Gy in 3 fractions in primary and metastatic liver cancers produces 1% of RILD [33]. In comparison of whole liver IR (> 80% of total liver volume) and focal liver IR (> 10% of total liver volume; 45 Gy), which mimics SABR in the clinic, we found 45 Gy of focal IR to mouse liver did not alter plasma FGL1 at 1 week or a month after IR. Otherwise, 30 Gy of liver IR showed higher FGL1 with high mortality (2 of 7) at 1 month after IR, and 15 Gy of liver IR showed increased FGL1 level at 1 week and then decreased levels in FGL1 at a month after IR. Our result also agree with previous reports that hepatic toxicity constitutes one of the most important consequences when the whole liver is exposed to radiation up to 30–35 Gy [18,19]. However, we could not clarify the relationship between threshold of FGL1 secretion and liver injury in this study. Therefore, further studies that clarify the relationship between FGL1 expression and liver toxicity with radiation volume and dose based on medical physics will be needed in the future. 

Although several trials to identify liver injury markers have been performed, no reliable biomarkers are available to date. The present study suggests a new perspective regarding the identification of liver injury biomarkers. In particular, we demonstrated the correlation of FGL1 expression and hepatocellular damage in human primary hepatocytes following irradiation. FGL1 was detected easily and showed good correlation with radiation dose in the acute or subacute phase in vivo and in vitro. These findings suggest that plasma FGL1 might serve as a potential biomarker for radiation-induced liver injury. Therefore, application of FGL1 for new biomarker may suggest new clues regarding the pathophysiology of liver injury following radiotherapy and may assist in stratifying people at risk of radiation accident.

## Supplementary Materials

The following are available online at https://www.mdpi.com/2073-4409/8/9/1042/s1, Figure S1: Target specificity of anti-FGL1 antibody. Validation of FGL1 antibody was conducted by Western blot to detect radiation-induced FGL1 in HHs with or without siRNA-induced knockdown, Figure S2: Measurement of secreted FGL1 from human hepatocytes to culture medium following irradiation. (A) FGL1 secretion into the culture medium was analyzed by ELISA at the indicated time. (B) Radiation dose dependency of secreted FGL1 in culture medium was detected by ELSA following 0–15 Gy of irradiation. Values represent the mean of three experiments in duplicate and are expressed as the means ± SEM (* *p* < 0.05), Figure S3: Expression of FGL1 in liver and pancreas tissue. At 3 days after 10 Gy of total body irradiation by Western blotting.
