# Supplementary material for: Plasma Fibrinogen-Like 1 as a Potential Biomarker for Radiation-Induced Liver Injury
Source: Cells. 2019 Sep 6;8(9):1042. doi: 10.3390/cells8091042 (PMC6770824; doi:10.3390/cells8091042)
Supplement: Supplementary file 1 [file cells-08-01042-s001.pdf]

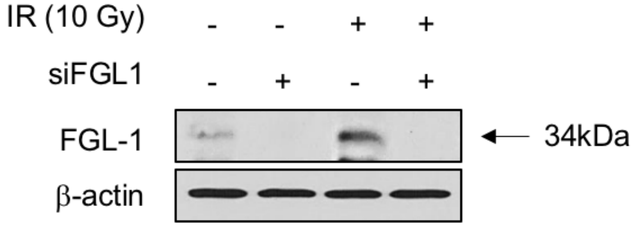

Supplementary Figure S1. Target specificity of anti-FGL1 antibody. Validation of FGL1 antibody was conducted by Western blot to detect radiation-induced FGL1 in HHs with or without siRNA-induced knockdown.

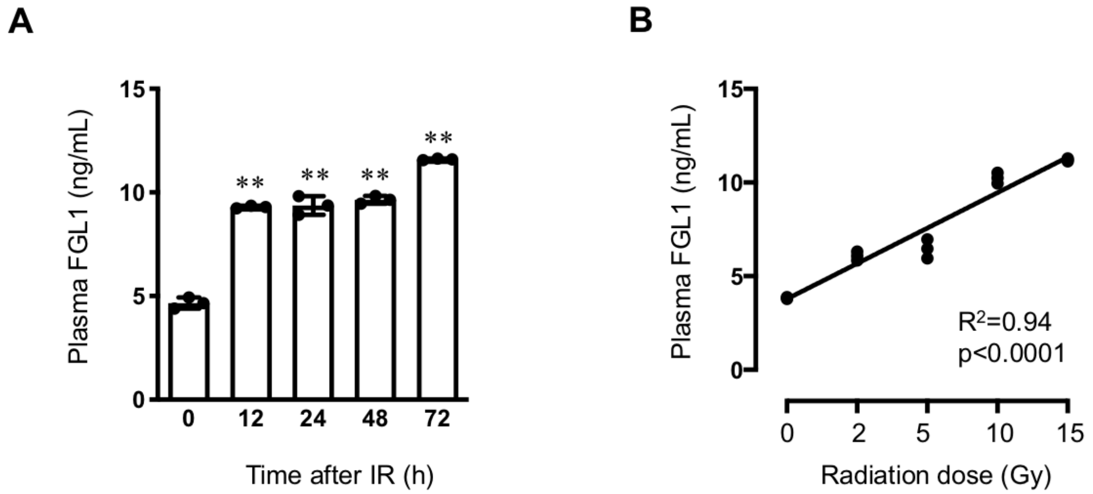

Supplementary Figure S2. Measurement of secreted FGL1 from human hepatocytes to culture medium following irradiation. (A) FGL1 secretion into the culture medium was analyzed by ELISA at the indicated time. (B) Radiation dose dependency of secreted FGL1 in culture medium was detected by ELSA following 0-15 Gy of irradiation. Values represent the mean of three experiments in duplicate and are expressed as the means ± SEM (*$p < 0.05$).

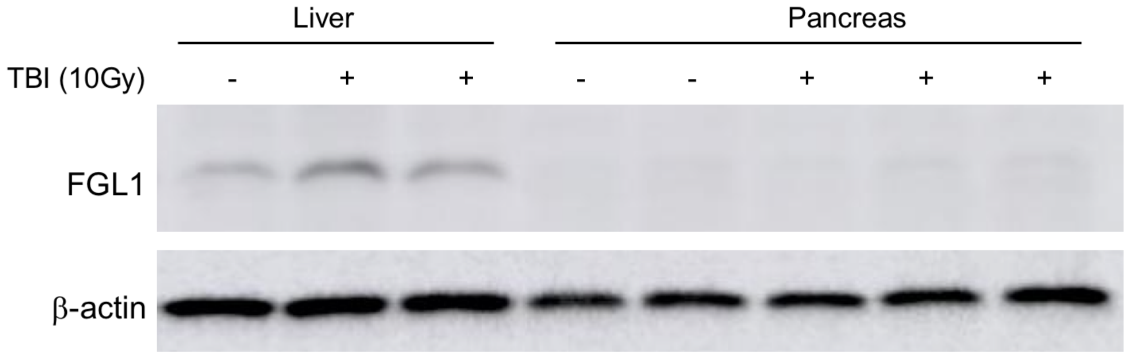

Supplementary Figure S3. Expression of FGL1 in liver and pancreas tissue. at 3 days after 10 Gy of total body irradiation by Western blotting.
